# The Biology of the Glutamatergic System and Potential Role in Migraine

**Published:** 2013-03

**Authors:** C. F. Gasparini, L. R. Griffiths

**Affiliations:** *Genomics Research Centre, Griffith Health Institute, Griffith University, Gold Coast Campus, Building G05, GRIFFITH UNIVERSITY QLD 4222, Australia*

**Keywords:** migraine with aura, migraine without aura, familial hemiplegic migraine, Glutamate, neurotransmitters, receptors, transporters, excitotoxicity

## Abstract

Migraine is a common genetically linked neurovascular disorder. Approximately ∼12% of the Caucasian population are affected including 18% of adult women and 6% of adult men (1, 2). A notable female bias is observed in migraine prevalence studies with females affected ∼3 times more than males and is credited to differences in hormone levels arising from reproductive achievements. Migraine is extremely debilitating with wide-ranging socioeconomic impact significantly affecting people’s health and quality of life. A number of neurotransmitter systems have been implicated in migraine, the most studied include the serotonergic and dopaminergic systems. Extensive genetic research has been carried out to identify genetic variants that may alter the activity of a number of genes involved in synthesis and transport of neurotransmitters of these systems. The biology of the Glutamatergic system in migraine is the least studied however there is mounting evidence that its constituents could contribute to migraine. The discovery of antagonists that selectively block glutamate receptors has enabled studies on the physiologic role of glutamate, on one hand, and opened new perspectives pertaining to the potential therapeutic applications of glutamate receptor antagonists in diverse neurologic diseases. In this brief review, we discuss the biology of the Glutamatergic system in migraine outlining recent findings that support a role for altered Glutamatergic neurotransmission from biochemical and genetic studies in the manifestation of migraine and the implications of this on migraine treatment.

## INTRODUCTION

Migraine is a complex debilitating neurovascular disorder, characterized by recurrent attacks of headache that differ in intensity, frequency and duration. The headache is often associated with an assortment of symptoms which can include nausea, emesis, photophobia, phonophobia, and occasionally, visual sensory disturbances. Migraine is estimated to affect approximately 12% of the Caucasian population (3) and shows a marked female preponderance (∼3:1). Migraine imparts significant mental, physical and social health implications to sufferers and their families.

Most migraine sufferers probably possess a number of genes that together contribute to susceptibility. Thus far genetic linkage and association studies have implicated a number of susceptibility genes and causative mutations that are of significant clinical relevance in migraine. However not all migraine genes have been uncovered and further research is necessary to determine the definitive molecular genetics of migraine. Numerous theories regarding the causes and underlying mechanisms that result in migraine symptoms have also been proposed.

The pathophysiology of this disorder implicates both neurological and vascular mechanisms. Current research suggests that the trigeminovascular system plays a significant role in migraine (4-6) due to its critical interaction with the meningeal vasculature and because various neurotransmitters, peptides, receptors and transporters are located in this system. The neurotransmitters implicated in migraine pathogenesis include: serotonin, dopamine and glutamate. An alteration in the balance of any of these neurological systems may lead to a higher susceptibility to migraine. Currently serotonin and dopamine remain the most studied neurotransmitter circuits in case-control association studies investigating polymorphisms in receptors, transporters, and enzymes of these systems (7, 8).

Migraine has a strong inherited component, and a large genetic study (9) suggests the involvement of glutamate pathways in migraine pathogenesis. Glutamate is implicated in elements of the pathophysiology of the disorder, including trigeminovascular activation, central sensitization and cortical spreading depression. Biochemical and pharmacological studies also support involvement of glutamate in migraine.

## GLUTAMATERGIC BIOLOGY

The pioneering work of Hayashi in 1954 established the physiological significance of glutamate as an excitatory neurotransmitter (10). Hayashi demonstrated glutamate’s role as a neurotransmitter in the CNS in experiments in dogs, monkeys and men, where injection of monosodium glutamate into the grey matter of the cortex was found to produce clonic convulsions (10).

Glutamate is a nonessential amino acid that does not cross the blood-brain barrier but must be synthesized inside neurons from local precursors (11). Glutamate is found in neurons of structures related to migraine pathophysiology, including the trigeminal ganglion, trigeminocervical complex and the thalamus (12). Glutamate has a number of metabolic fates in brain, including oxidation via the TCA cycle for energy, incorporation into proteins, and formation of glutamine, γ-aminobutyric acid (GABA), and glutathione (13). The brain contains large amounts of glutamate the majority of which is stored intracellularly (13). Glutamate is cycled continuously between neurons and glial cells in what is known as the glutamate-glutamine cycle under normal conditions (13). There are a number of enzymes involved in this cycling as outlined in Table 1 that are important for the metabolism of Glutamate and are subject to regulation.

**Table 1:** Enzymes involved in glutamate cycling

Enzymes

PDH, pyruvate dehydrogenase
PAG, phosphate activated glutaminase
mME, mitochondrial malic enzyme
GAD, glutamic acid decarboxylase
GS, glutamine synthetase
PC, pyruvate carboxylase
cME, cytosolic malic enzyme
AAT, aspartate aminotransferase
GDH, glutamate dehydrogenase

The neuronal/glial cell interface where glutamate cycling occurs contains glutamate receptors, which are responsible for signal input; plasma glutamate transporters, which are responsible for signal termination and vesicular glutamate transporters for signal output through exocytic release (14). These are the biological constituents of the Glutamatergic system. The Glutamatergic system of the brain is one of the two major amino acid systems, GABA being the other. The glutamate system is a fast-signalling system that is very important for information processing in neuronal networks of the neocortex and hippocampus.

## GLUTAMATE RECEPTORS

Glutamate is the most abundant excitatory neurotransmitter in the brain; it is critical to the communication of nerve cells with one another in practically every circuit in the nervous system. Glutamate communicates in this circuit via two main subtypes of membrane receptors, ionotropic and metabotropic. The family of ionotropic receptors is divided into three groups, referred to as N-methyl-D-aspartate (NMDA) receptors, α-amino-3-hydroxy-5-methyl-4-isoxazole propionate (AMPA), and kainate (KA) receptors on the basis of DNA sequence similarity and their activation by different pharmacologic agonists (Table 2) (15). The family of metabotropic receptors mGluRs consists of at least eight receptor types also divided into three groups (Table 3).

**Table 2:** Ionotropic glutamate receptors

Receptor Type	Gene	Chromosome (Human)

AMPA	GRIA1	5q33
AMPA	GRIA2	4q32-33
AMPA	GRIA3	Xq25-26
AMPA	GRIA4	11q22-23
Kainate	GRIK1	21q21.1-22.1
Kainate	GRIK2	6q16.3-q21
Kainate	GRIK3	1p34-p33
Kainate	GRIK4	11q22.3
Kainate	GRIK5	19q34.3
NMDA	GRIN1	9q34.3
NMDA	GRIN2A	16p13.2
NMDA	GRIN2B	12p12
NMDA	GRIN2C	17q24-q25
NMDA	GRIN2D	19q13.1qter
NMDA	GRIN3A	9q31.1
NMDA	GRIN3B	19p13.3
Orphan	GRID1	-
Orphan	GRID2	4q22

**Table 3:** Metabotropic glutamate receptors

Receptor Type	Gene	Chromosome (Human)

mGluR_1_	GRM1	6q24
mGluR_5_	GRM5	11q14.3
mGluR_2_	GRM2	3p21.2
mGluR_3_	GRM3	7q21.1-q21.2
mGluR_4_	GRM4	6p21.3
mGluR_6_	GRM6	5q35
mGluR_7_	GRM7	3p26-p25
mGluR_8_	GRM8	7q31.3-q32.1

The difference between ionotropic and metabotropic receptors is that ionotropic receptors are ligand-gated ion channels, while the metabotropic receptors (Table 3) (mGluRs) are G-protein coupled receptors, and their activation is coupled to an intracellular biochemical cascade leading to modulation of second messengers (15). They are strategically situated on several cell types converging on the glutamate synapse: pre and post-synaptic neurons, astrocytes (a type of glial cell), and nearby inhibitory neurons that use (GABA) (14). Glutamatergic receptors have been extensively studied in neurologic diseases.

An interesting genetic mechanism pertinent to a discussion of GRIA receptors is that the GluR2 subunit undergoes RNA editing at a specific point known to affect the Ca^2+^ permeability of the channel (16). The position that regulates the Ca^2+^ permeability is the Q/R site and this is edited by the ADAR2 enzyme. This gene performs the RNA editing function necessary for the maturation of glutamate and serotonin receptor transcripts and therefore plays an important role in the regulation and fine tuning of Glutamatergic neurotransmission (17). ADAR enzymes are generated in humans by three independent genes ADAR-1-3 located on chromosomes 1, 21, and 10 (18). There is some evidence of involvement of another RNA editing gene ADARB2 from a pedigree based GWAS in the Norfolk Island population (19).

RNA editing is a physiologically important process that affects several features of the receptors, including kinetics, subunit assembly and cell-surface expression and if editing is prevented the channels become permeable to Ca^2+^ causing neuronal cell death (16, 20). Other neuronal genes affected by A-to-I RNA editing include the glutamate receptor subunits GluR-3, -4, -5, and -6 where RNA editing regulates gating and kinetic properties of the ion channels, the 5-HT2C serotonin receptor subtype where editing is known to regulate G-protein coupling functions of the receptor, and the K(V)1.1 potassium channel where editing regulates channel inactivation (16).

RNA editing genes have been suggested as candidate genes for complex neurological disorders such as epilepsy, depression and schizophrenia and amyotrophic lateral sclerosis (ALS). The role of adenosine deaminase RNA editing of glutamate and serotonin receptor transcripts is further exemplified by the disorder, amyotrophic lateral sclerosis (ALS) (MIM 105400). In amyotrophic lateral sclerosis (ALS) editing of mRNA encoding the GluR2 subunit of glutamate AMPA receptors in spinal motor neurons is defective and interferes with normal functioning of the glutamate receptors. Knockout mice in RNA editing genes are lethal implying that this mechanism is essential for survival and dysregulation could potentially affect Glutamatergic function. Genes involved in RNA editing are candidate genes to investigate due to the impact that this mechanism has on the Glutamatergic system.

## GLUTAMATE RECEPTOR STRUCTURE

X-ray crystal structures have produced 3D images that have helped understand the structural basis of glutamate receptors revealing which domains are involved in binding to agonists and antagonists and to study the effect of mutations on protein conformation to understand how this relates to neuronal function in neurological disease. So far a number of structures have been described for a membrane-spanning tetrameric glutamate receptor as well as in complex with various agonists, antagonists, and modulators (21-27). These experiments have been possible due to advances in molecular modelling and structural biology techniques. These data, along with functional and biochemical experiments, have begun to define the relationship between receptor structure and function and have contributed to understanding the neurotransmitter binding mechanisms at the synapse.

## GLUTAMATE TRANSPORT

Glutamate concentrations in the extracellular space under normal conditions are kept low and tightly controlled by EAAT transporters present in both nerve endings and surrounding glial cells. Perturbations to this regulatory system can have deleterious effects such as excess release of glutamate, which can induce hyperexcitability in post-synaptic neurons to the point of excitotoxicity and cell death (14). The transport process is considered to be primarily responsible for the termination of neurotransmitter action of glutamate and the prevention of neuronal damage from excessive activation of glutamate receptors (28).

There are two transport systems: the plasma GluTs, that are responsible for signal termination; and the vesicular GluTs for signal output through exocytic release. Five different ‘high-affinity’ glutamate (excitatory amino acid) transporters have been identified by cloning including Excitatory Amino Acid Transporters located in the plasma membrane, EAAT1, EAAT2, EAAT3, EAAT4 and EAAT5. The vesicular GluTs are crucial for the storage of Glu in synaptic vesicles, three isoforms exist VGLUT1, VGLUT2, VGLUT3 (29). Genetic mutations in receptors, transporters and enzymes involved in glutamate metabolism could contribute to derailed function of the Glutamatergic system and these are key candidates for future genetic studies.

## GLUTAMATE TOXICITY

Observations by Lucas and Newhouse in 1957 (30) described the toxic properties of glutamate by injecting monosodium glutamate into the neurons of the retina from newborn mice. The term “glutamate excitotoxicity” was introduced by Olney (31) to describe the toxic action of glutamate which caused neuronal cell death. Excitotoxicity is the pathological process by which nerve cells are damaged and killed by excessive stimulation of neurotransmitters such as glutamate and similar substances and is considered a normal physiological response to CNS insult (32).

Excitotoxicity may be involved in stroke, traumatic brain injury and neurodegenerative diseases of the central nervous system (CNS) such as Multiple sclerosis, Alzheimer’s disease, Amyotrophic lateral sclerosis (ALS), Fibromyalgia, Parkinson’s disease, and Huntington’s disease. The mechanism of this excitotoxicity is thought to be due to the entry of high levels of Ca^2+^ ions into the cell from over stimulation with glutamate (33). Ca^2+^ influx into cells activates a number of enzymes, including phospholipases, endonucleases, and proteases such as calpain that go on to damage cell structures such as components of the cytoskeleton, membrane and DNA (34). This is thought to be the mechanism that leads to neuronal cell death. Excessive activation of GluRs during stress to the brain, such as ischemia, head trauma, and epileptic seizures leads to the death of central neurons.

## MIGRAINE MOLECULAR GENETICS

Our understanding of ‘migraine genetics’ is an evolving subject due to its relative novelty in this disease system. There have been some successes such as the recent identification of a functional mutation in the KCNK18 gene. The KCNK18 gene encodes TRESK, a potassium channel that is part of the subfamily K member 2 (K2P) channels, which are expressed throughout the central nervous system, including the trigeminal ganglion neurons (35). A number of mutations in this gene were identified using a candidate gene approach and functional analysis, in a large cohort of both case-control individuals and multi-generational families by directly Sanger sequencing the DNA of a panel of 110 unrelated migraine probands (36). The most notable variant identified was a frameshift mutation (F139WfsX24), which segregated perfectly in a family affected with typical MA. The TRESK is involved in pain pathways and regulates neuronal excitability and is an exciting discovery because it is the first genetic mutation to be linked to common migraine (37).

The most established molecular knowledge of migraine comes from mutations in the three genes for familial hemiplegic migraine (FHM) – CACNA1A, ATP1A2 and SCN1A (38-40). Familial hemiplegic migraine (FHM) is a rare form of migraine distinguished from classical migraine, by a prolonged aura and a more genetically determined component. Transcription of the three causal genes results in protein products that assemble to make heteromeric ion channels in the plasma membranes of cells. Mutations in FHM genes are thought to increase neuronal excitability and reduce the threshold for cortical spreading depression. Disorders attributed to mutations in ion channel genes have been classified as ‘channelopathies’. More than 40 different channelopathies have been identified, affecting all electrically excitable tissues: brain, peripheral nerve, skeletal muscle, smooth muscle, and heart (41, 42). FHM has been included in this list following the discovery that two of its causative genes encode voltage-gated ion channels and as a result has strengthened the idea of migraine as a disorder of ‘neuronal excitability’.

Ion channel genes play a critical role in normal functioning of the central nervous system where they control important biological functions including the release of neurotransmitters, hormones and muscle contraction (43). Ion channels are macromolecular protein complexes that span the membrane lipid bilayer and facilitate the movement of ions across this hydrophobic barrier that separates the cytoplasm from the extracellular space or from intracellular organelles (41). Ion flux through channels is the source of the electric current to regulate the membrane potential and thus is the fundamental basis for cellular electrical excitability. Minute alterations in the amino acid sequence or expression of these ion channels from genetic mutations can result in changes affecting the biophysical properties of the channel such as permeation and gating. This is of significant consequence to neurological, retinal, cardiac, and muscular tissues that rely on fast signal transmission and gross pathological changes can lead to serious chronic disorders (43).

## GLUTAMATE GENETIC STUDIES

Genetic association studies have mostly investigated variants in serotonin and dopamine receptor genes. Fewer studies have been done in relation to the genetics of the Glutamatergic system in migraine. The first genetic evidence of a link between migraine and glutamate was provided by Formicola *et al.*, 2010 who found a positive association in 3 SNPs in the AMPA receptors GRIA1 and GRIA3 in an Italian population (44). The ionotropic AMPA (GRIA) receptors are comprised of 4 subunits coded by the glutamate receptor 1 to 4 genes at chromosomal loci 5q33, 4q32, Xq24 and 11q24 respectively (15). The AMPA receptor proteins are products of separate genes that arrange to form ligand-gated ion channels in the plasma membrane permeable to Na^+^, K^+^ and Ca^2+^ (45). The four domains are arranged in a tetrameric structure to form a transmembrane aqueous pore (46). Two SNPs in GRIA1 (5q33.2, rs548294 MO allelic P=0.008, rs2195450 MA allelic P=0.0005) and 1 SNP in GRIA3 (rs3761555 MA Females allelic P=0.003) showed a positive association with migraine (44). The remaining subfamilies of kainate (KA) and N-methyl-D-aspartate (NMDA) and metabotropic receptors are yet to be investigated in migraine association studies. It is noteworthy that genes of the Glutamatergic system have also been investigated in association studies of other disorders like schizophrenia given their neuronal role.

The role of glutamate in migraine pathology has gained momentum with the recent discovery of a plausible genetic risk variant implicated in a large-scale genome wide association study of migraine (9). The GWAS published by Antilla, *et al*., 2010 found the genetic risk variant to be located between the genes MTDH and PGCP both of which are in pathways thought to regulate glutamate accumulation in the synaptic cleft. The variant affects MTDH gene expression and thereby indirectly regulates the expression of the glutamate transporter gene EAAT2, encoding a major glutamate transporter in the brain. The effect of this marker was consistently stronger in the migraine with aura only groups than other migraine subgroups with P=5.38 × 10^-9^ and odds ratios ranging between 1.21 and 1.33. Due to the role of these two genes in glutamate homeostasis, it seems that complementary pathways such as the glutamate system could fasten mendelian channelopathies with pathogenesis of common forms of migraine (9) (Table 4).

**Table 4:** Glutamate transporters

Transporter Type	Gene	Chromosome (Human)

EAAT1	SLC1A3	5p13
EAAT2	SLC1A2	11p13
EAAT3	SLC1A1	9p24
EAAT4	SLC1A6	19p13.12
EAAT5	SLC1A7	1p32.3
VGLUT1	SCL17A7	9q13.33
VGLUT2	SCL17A6	11p14.3
VGLUT3	SCL17A8	12q23.1

In addition to glutamate receptors, glutamate transporters can contribute to neurologic dysfunction and could be useful molecular targets for treatment. EAAT transporters play a key role in the regulation of extracellular glutamate levels in the central nervous system where they protect neurons from excitotoxic damage. A number of studies have implicated EAATs in the pathophysiology of stroke, epilepsy, amyotrophic lateral sclerosis (ALS), Huntington Disease, HIV-associated dementia, malignant glioma, and other neurologic disorders (47). Jen *et al.*, 2005 examined a *de novo* mutation in the transporter EAAT1, in a patient with episodes of ataxia, migraine, hemiplegia and seizures (48). The authors concluded that the missense mutation P290R contributed to neuronal hyperexcitability through decreased transporter function resulting in the hemiplegia and other neurological symptoms.

Another study of the EAAT1 gene by de Vries *et al.*, 2009 in patients with Episodic Ataxia (EA) identified a novel pathogenic mutation C186S in one patient. The mutated EAAT1 protein showed severely reduced uptake of glutamate (49). The severity of EA symptoms appears to be correlated with the extent of glutamate transporter dysfunction. The syndrome was designated EA6 and shares overlapping clinical features with EA2, which is caused by mutations in CACNA1A the FHM locus.

An association study by Shin *et al.*, 2011 evaluated the contribution of polymorphisms in the EAAT2 transporter and found no direct association between this genetic factor and migraine (50). The EAAT2 transporter has been investigated in association with a number of other disorders given it is responsible for up to 90% of all glutamate transport in adult tissue (51-53). A study by Mallolas and colleagues has found a novel and highly prevalent polymorphism in the promoter of the EAAT2 glutamate transporter gene. This polymorphism was associated with higher and maintained plasma glutamate concentrations as well as with higher frequency of neurological deterioration in patients with acute hemispheric stroke. In conclusion, this study has revealed a novel functional polymorphism in the EAAT2 promoter region and a pattern of regulation that decreases promoter activity.

Alterations in the function or expression of components of this system may be involved in migraine susceptibility. Further research into the Glutamatergic system is necessary to ascertain its role in migraine aetiology.

## GLUTAMATERGIC SYSTEM AND CLINICAL IMPLICATIONS

Pharmacological compounds capable of modulating glutamate receptors have helped untangle the functional role of glutamate receptor family members and present promising targets for the treatment of migraine. Considerable scope however remains for the development of novel ligands that will encompass the family of glutamate receptors. Currently the most promising compounds reported in the literature include: topiramate, ketamine and memantine. Several types of drugs, like generic beta blockers, calcium channel blockers, tricyclic antidepressants and anti-epileptic drugs are given to prevent migraines, these are not always effective in all patients. Targeting the glutamatergic system offers a novel approach to treatment in view of the limited efficacy of existing drugs.

Topiramate is a derivative of the naturally occurring monosaccharide D-fructose that was originally developed as an anticonvulsant and is recognized as an effective medication for migraine prevention (54). Topiramate is a glutamate receptor antagonist within the trigeminothalamic pathway. Topiramate has several actions which are relevant, including the blockade of Na^+^ and Ca^2+^ channels, enhancement of GABA activity, and blockade of ionotropic glutamate receptors (54).

A few other compounds Memantine, Ketamine and ADX10059 are drugs that act on glutamate signalling through NMDA receptors (55, 56). Memantine is a moderate-affinity noncompetitive antagonist at glutamatergic N-methyl-D-aspartate (NMDA) receptors (55). Preclinical experiments and small scale studies in migraineurs with these drugs have been useful in demonstrating the role that NMDA receptors play in the "migraine circuit," a positive feedback loop that generates the symptoms of a migraine attack.

## CONCLUSION

Migraine is a disabling costly brain disorder, with hypothesised involvement of neurotransmitters. The major excitatory neurotransmitter of the brain, glutamate and the receptors, upon which it acts, are intimately involved in trigeminovascular nociceptive processing. Given the importance of the Glutamatergic system and its involvement in biological processes involved in the brain, genes of this system remain candidates for further investigation. The role of glutamate antagonists in the treatment of migraine is added evidence of a role for glutamate in migraine. Further research is required to elucidate the mechanism through which GRIA genes may contribute to migraine and to determine if other unknown mutations in components of this system may be contributing to the migraine phenotype.

Genetic characterization of migraine as a disorder is making steady progress with an increasing number of genomic susceptibility loci now identified. The data and ideas presented above have lent strong support implicating glutamate biology in migraine pathophysiology at the turn of the 21^st^ century. The genetic studies are small and more data is needed to draw any solid conclusion about involvement of Glutamatergic genes in migraine. Nonetheless the genetic evidence is growing with association, linkage and GWAS results bringing to light new variants and genomic regions.

The identification of these migraine specific loci will contribute to more specific pharmacotherapeutics for the patient. Genetic variation greatly affects patient response to treatment and further insight could lead to more individualized treatments leading to better tolerability.
